# Aceclofenac fast dispersible tablet formulations: Effect of different concentration levels of Avicel PH102 on the compactional, mechanical and drug release characteristics

**DOI:** 10.1371/journal.pone.0223201

**Published:** 2020-02-12

**Authors:** Riffat Yasmin, Muhammad Harris Shoaib, Farrukh Rafiq Ahmed, Faaiza Qazi, Huma Ali, Farya Zafar

**Affiliations:** Department of Pharmaceutics, Faculty of Pharmacy and Pharmaceutical Sciences, University of Karachi, Karachi, Pakistan; Aristotle University of Thessaloniki, GREECE

## Abstract

The objective of this study was based on the formulation development of fast dispersible Aceclofenac tablets (100 mg) and to evaluate the influence of pharmaceutical mixtures of directly compressible Avicel PH102 with Mannitol and Ac-di-sol on the compressional, mechanical characteristics and drug release properties. Fast dispersible Aceclofenac formulations were developed by central composite design (CCD). Among them the best possible formulation was selected on the basis of micromeritic properties, appropriate tablet weight and disintegration time for further study. Tablets were directly compressed using manual hydraulic press with a compressional force ranging from 7.2 to 77.2 MN/m^2^. Pre and post compression studies were performed and the compressed formulations (FA-FF) were assessed for different quality tests. The Heckel and Kawakita equations were applied for determination of compressional behavior of formulations. The quality attributes suggested that formulation (FB) containing avicel PH 102 (20%), mannitol (25%) and ac-di-sol (3%) as best optimized formulation showing better mechanical strength i.e. hardness 35.40 ± 6.93N, tensile strength 0.963 MN/m^2^, and friability 0.68%. Furthermore, compressional analysis of FB showed lowest P_Y_ value 59.520 MN/m^2^ and P_k_ value 1.040 MN/m^2^ indicating plasticity of the material. Formulation FB disintegrated rapidly within 21 seconds and released 99.92% drug after 45 min in phosphate buffer pH 6.8. Results of drug release kinetics showed that all formulations followed Weibull and First-order models in three different dissolution media. Avicel PH102 based formulation mixture exhibit excellent compactional strength with rapid disintegration and quick drug release.

## Introduction

Advancement in tablet manufacturing technology has offered viable dosage alternatives for those patients who are facing problem related to compliance with conventional dosage forms. One such alternative dosage form is fast dispersible tablets [1]. These tablets are of two types: first type is taken in mouth without water to disintegrate rapidly or disperse readily and the second type of tablets form dispersion or solution in water to be taken by patients [2, 3]. These tablets are usually developed by direct compression method. Aceclofenac is a Cycloxygenase inhibitor having analgesic and anti-inflammatory activity. Due to its short half-life (4hr) and twice daily dose, it is considered a suitable candidate for fast dispersible tablets [4, 5].

Avicel^®^, the first commercialized brand of microcrystalline cellulose (MCC), is introduced by FMC Corporation as a direct compression tableting ingredient. MCC is a partially depolymerized cellulose that is obtained as pulp by mineral acid treatment of alpha cellulose type lb of fibrous plant material. Cellulose is the most abundant natural polymer having linear chains of b-1, 4-D anhydroglucopyranosyl units. Pharmaceutical MCC is most commonly obtained from wood where cellulose chains are packed in layers held together by strong hydrogen bonds and lignin (cross-linking polymer). The primary particles of all MCC types (101, 102 and 200) are about 50μm but the difference in the larger 2%, 2% aggregated particle numbers. Type 102 has a median particle size of about 100μm indicating adequate flow properties for successful tableting. MCC deforms plastically and maximizes the interparticle bonding area during compression. It forms strong and cohesive compacts even under low compression pressure due to the formation of numerous hydrogen bonds. Tableting is further enhanced by mechanical interlocking of elongated and irregularly shaped particles [6].

Formulations designed for fast disintegration require sugar-based diluents to impart pleasant taste, water solubility, and ability to mask the bitterness of medicament. Mannitol was used in this study to improve the mouthfeel of fast dispersible tablets [7]. A combination of avicel PH102 with water soluble mannitol exhibits shorter disintegration time and increased water solubility of mannitol. It may lead to the formation of pores in the tablets matrix, causing capillary action for water permeation into the tablet matrix; resulting in fast disintegration [8].

Ac-Di-Sol (i.e. super disintegrant) exerts its action by wicking and swelling of the tablet. The porous nature of Ac-Di-Sol provides access for water diffusion in tablets, resulting in wicking and the faster disintegration of tablets. It is recommended to be used in 0.5–3% concentration for directly compressible tablets [9–12].

During tablet manufacturing, compression of powder shows a reduction in the volume of powder bed by the application of compressional pressure in a confined space. As a result, there is a formation of strong inter-particle bonds that produce a compact mass and built inherent strength in the compact to increase the overall mechanical strength of the dosage unit. This analysis is significant in understanding the behavior of poorly compactable powders during the manufacturing of the tablets[13]. Heckel and Kawakita equations have been largely employed to understand this relationship between applied compressional pressure and mechanical strength of the compact.

The aim of the present study was to prepare fast dispersible Aceclofenac tablets and to evaluate the compressional behavior of the newly developed tablets using Heckel and Kawakita analysis.

Formulations were designed by Central Composite Design (CCD) using varying concentrations of avicel PH102, mannitol, and ac-di-sol. All the formulations were developed by direct compression method.

## Materials

Aceclofenac was gifted by Sami Pharmaceutical (Pvt.) Limited. Avicel PH-102, mannitol, ac-di-sol, aspartame, talc, and vanilla flavor were purchased from FMC Corporation, USA.

## Methods

### Experimental design

Twenty fast dispersible Aceclofenac tablet formulations (100mg) were designed with the help of Central Composite Design using Design Expert^®^10.0 software (Stat–Ease, Inc, Minneapolis, MN 55413, USA). The selected independent variables were avicel PH102 (20–35%), mannitol (10–25%) and ac-di-sol (0.5–3%). Excipients such as aspartame, vanilla flavor and talc were used at a fixed concentration i.e. 2%, 1%, and 2% respectively. Disintegration time, percentage friability and hardness of formulations were selected as the dependent variables. The run type was arranged and recoded as F1 = FA, F2 = FB, …………. F15 = FO. The composition of each formulation in % and mg is mentioned in Table 1. There was six center points out of 20 formulations (i.e. F3 = center point formulation) with the same composition. These six formulations showed almost the same responses (disintegration time, friability and hardness) therefore results of only one center point formulation (i.e. F3 = Centre point formulation) is mentioned in proceeding text.

**Table 1:** Composition of fast dispersible Aceclofenac formulations using central composite design.

Ingredients amount in %									Ingredients amount in mg							
**Run**	**Formulation Codes**	**Avicel PH102**	**Mannitol**	**Ac-di-sol**	**Aspartame**	**Talc**	**Flavor**	**Drug**	**Avicel PH102**	**Mannitol**	**Ac-di-sol**	**Aspartame**	**Talc**	**Flavor**	**Drug**	**Tablet weight**
		**X1 (%)**	**X2 (%)**	**X3 (%)**	**(%)**	**(%)**	**(%)**	**(%)**	**X1 (mg)**	**X2 (mg)**	**X3 (mg)**	**(mg)**	**(mg)**	**(mg)**	**(mg)**	**(mg/tab)**
**F1**	**FA**	20.00	25.00	0.50	2	2	1	49.50	40.80	51.00	1.02	4.08	4.08	2.04	100	203.02
**F2**	**FB**	20.00	25.00	3.00	2	2	1	47.00	42.55	53.00	6.36	4.25	4.25	2.12	100	212.53
*F3	**FC**	27.50	17.50	1.75	2	2	1	48.25	57.20	36.45	3.64	4.16	4.16	2.08	100	207.69
**F4**	**FD**	27.50	17.50	3.39	2	2	1	46.61	58.91	37.45	7.10	4.28	4.28	2.14	100	214.16
**F5**	**FE**	35.00	10.00	0.50	2	2	1	49.50	71.42	20.40	1.02	4.08	4.08	2.04	100	203.04
**F6**	**FF**	35.00	10.00	3.00	2	2	1	47.00	74.20	21.20	6.36	4.25	4.25	2.12	100	212.38
**F7**	**FG**	20.00	10.00	0.50	2	2	1	64.50	31.24	15.62	0.78	3.12	3.12	1.56	100	155.44
**F8**	**FH**	20.00	10.00	3.00	2	2	1	62.00	32.20	16.10	4.83	3.22	3.22	1.61	100	161.18
**F9**	FI	27.50	07.63	1.75	2	2	1	58.12	47.52	13.18	3.02	3.45	3.45	1.72	100	172.34
**F10**	FJ	17.63	17.50	1.75	2	2	1	58.12	30.46	30.24	3.02	3.45	3.45	1.72	100	172.34
**F11**	FK	35.00	25.00	3.00	2	2	1	32.00	109.37	78.00	9.36	6.25	6.25	3.12	100	312.35
**F12**	FL	27.50	27.37	1.75	2	2	1	38.38	72.05	71.70	4.58	5.24	5.24	2.62	100	261.43
**F13**	FM	35.00	25.00	0.50	2	2	1	34.50	102.90	73.50	1.47	5.88	5.88	2.94	100	292.57
**F14**	FN	37.37	17.50	1.75	2	2	1	38.38	98.00	45.89	4.58	5.24	5.24	2.62	100	261.57
**F15**	FO	27.50	17.50	0.10	2	2	1	49.89	50.09	31.88	0.191	3.64	3.64	1.82	100	191.26

*F3 = Centre point formulation

Response surface methodology (RSM) was used to explore the interaction of avicel PH102, mannitol and ac-di-sol to establish the appropriate amount of excipient for optimized fast dispersible formulation. On the basis of the fit summary, ANOVA and multiple correlation coefficient appropriate model was selected.

### Pre-compression studies

#### Assessment of powder densities and flow characteristics

Bulk density of all formulations was evaluated using a measuring cylinder. The cylinder’s weight was tare to zero and then filled with certain amount of formulation blend and reweighed. The following formula was used to determine the bulk density (g/cm^3^).

ρbulk=Massbulkvolume Eq 1

Where ρ_bulk_ is the bulk density, and bulk volume is the initial volume occupied by the formulation blend in the cylinder.

The cylinder was tapped 100 times and reduction in formulation bed’s volume was recorded as tapped volume and the following formula was used to calculate the tapped density (g/cm^3^).

ρtap=Masstappedvolume Eq 2

Formulation blends were also assessed by Hausner’s Ratio (HR) and angle of repose (ϴ) using the following equations [14]:

$${Hausner}^{\prime }s\;Ratio=\frac{\rho_{tapped}}{\rho_{bulk}}$$ Eq 3

$$\theta ={tan}^{-1}\frac{2h}{D}$$ Eq 4

In the above equation *θ* is the angle of repose, ‘h’ is the height of powder heap and ‘D’ is the diameter of the heap formed. True density (ρ_t_) of all powder blends were determined by *liquid displacement method* using xylene as displacement liquid with the help of a pycnometer. The difference between empty pycnometer (W) and xylene filled pycnometer (W_1_) was calculated as the weight of xylene (W_2_). Approximately 2 g sample of each formulation was weighed (W_3_) and transferred to the pycnometer along with xylene and weighed again (W_4_). True density (g/cm^3^) was calculated using the following equation [15]:

Weight of empty pycnometer = W

Weight of pycnometer filled with xylene = W_1_

Weight of xylene W_2_ = W_1_-W

Weight of formulation blend = W_3_

Weight of pycnometer filled with xylene and formulation blend = W_4_

$$\rho_{t}=\frac{\left(W_{2}\times W_{3}\right)}{50\times (W_{3}-W_{4}+W_{2}+W)}$$ Eq 5

#### Compression of powder blends

All the ingredients were weighed and passed through 20 mesh sieve separately and mixed for six minutes (optimized mixing time) by tumbling in a polybag. After mixing all the ingredients, talc was added and mixed for a further five minutes. Blends were compressed by manual filling of the die cavity. Tablets were compressed by applying varied compressional pressure 7.72, 23.16, 30.88, 38.0, 46.32, 54.04, 61.76, 69.48 and 77.2 MN/m^2^) using a manual hydraulic tableting machine (locally manufactured) fitted with pressure gauge. Tablets were stored in a desiccator for 24 h over silica gel for elastic recovery and hardening.

#### Evaluation of physicochemical parameters of Aceclofenac formulations

Compressed tablets were weighed and their thickness and diameter were accurately measured with digital vernier caliper (Digital Caliper: Seiko brand). Tablet hardness was determined by using hardness tester (OSK Fujiwara, Ogawa Seiki Co. Ltd., Tokyo, Japan). Tensile strength of round flat-faced tablets was calculated using the given equation [16]:

$$T=\frac{2F}{\pi DH}$$ Eq 6

Where, F (N) is the crushing load applied on tablets (i.e. hardness), ‘H’ is the thickness (mm) and ‘D’ is the diameter (mm) of tablets. Percentage friability was determined using Roche type Friabilator (H. Jurgens Gmbh H and Co- Bremen, D2800, Germany) with the help of following formula:

$$F=\frac{W_{o}-W_{t}}{W_{o}}\times 100$$ Eq 7

Where W_o_ is the initial weight and W_t_ is the final weight of tablets after 100 rotations. For the disintegration test of fast dispersible tablets, 900 mL distilled water was maintained at 37°C. Tablets were disintegrated in seconds [17]. The assay assessment was carried out using UV-Spectrophotometer (UV-1800 Shimadzu Corporation Kyoto, Japan) at 274 nm absorbance [18, 19]. Similarly, the dissolution test was performed using 0.1N HCI, phosphate buffer pH 4.5 and pH 6.8.

#### Evaluation of compressional behavior

*Heckel analysis*. Mathematically Heckel equation is expressed as follows:

ln[11−ρr]=KP+A Eq 8

This equation is used to relate the powder bed’s relative density (ρ_r_) to the applied pressure during compression which is the ratio of apparent density (ρ_A_) of the tablet and the true density (ρ_T_) of powder blend:

ρr=ρAρT Eq 9

The apparent density ρ_A_ of tablet is calculated using the weight (g), radius (cm) and thickness (cm) of the tablet, whereas true density of the powder blend is determined by liquid displacement method (g/cm^3^).

ρA=Weightoftabletπr2h Eq 10

By plotting the values of ln[1(1−ρr)] and applied pressure ‘P’, linear portion of the plot provides the values of slope and intercept (i.e. K and A). The value of K determines the mean yield pressure (1/K = P_Y_). This mean yield pressure P_Y_ indicates the plasticity of material under compression. Smaller the value of 1/K, higher the plasticity of material. Using the value of intercept ‘A’ densification of powder at different stages (i.e. D_o_, D_A,_ and D_B_) is determined [15, 20, 21]. The value of D_o_ (relative density = bulk density / true density) shows the powder densification at die filling stage. Since it measures the packing characteristics of powder, thus high value of D_o_ indicates high dense packing of powder blend [22, 23]. The D_B_ value indicates densification of powder when it is compressed and particles show movement and re-arrangement. Magnitude of rearrangement based on the theoretical point of densification at which deformation of particles started [24]. The value of D_B_ is calculated as follows:

$$D_{B}=D_{A}-Do$$ Eq 11

The D_A_ value is the final compact densification (D_A_ = ρ_r_) and it is calculated from intercept ‘A’ of the Heckel plot:

$$D_{A}=1-e^{-A}$$ Eq 12

#### Kawakita equation

Kawakita equation describes the compressional behavior of powders, either by tapping or from continuous compression experiments [25]. This equation explains the relationship between the degree of volume reduction ‘C’ upon the application of compressional pressure ‘P’. The linear expression of the Kawakita equation is given below:

$$\frac{P}{C}=\frac{P}{a}+\frac{1}{ab}$$

or

$$C=\frac{\left(V_{O}-V_{P}\right)}{V_{O}}=\frac{abP}{\left(1+bP\right)}$$ Eq 13

Where V_o_ is the initial bulk volume of the powder, V_p_ is the volume of the powder after compression, a is the total volume reduction for the powder bed or minimum porosity before compression and ‘b’ is the powder’s plasticity [26]. A graph of $\frac{P}{C}$ versus P was plotted for all formulations and constants a and b were determined from slope and intercept of the plot. Reciprocal of slope produced the value of a (i.e. a = 1/slope) and reciprocal of the intercept yielded, ab (i.e. ab = 1/Intercept).

b=ab−a

Reciprocal of b is a pressure (P_K)_ which reduced the thickness of powder bed 50%.

#### Evaluation of in-vitro release behavior

Six tablets of each test (FA-FH) and immediate release marketed (reference) formulations were placed in the dissolution apparatus (USP Apparatus-II: Paddle Stirring Element) containing 900 mL of dissolution media at 37± 0.5°C at 50 rpm. Multiple points sampling was conducted in different dissolution media i.e. 0.1N HCl, phosphate buffer pH 4.5 and 6.8. 10 ml sample was withdrawn at 5, 10, 15, 20, 30, 45, 60, 90 and 120 min interval and substituted with fresh 10 ml of same solution. Each test and reference solutions were diluted, filtered and analyzed spectrophotometrically at 274nm [19].

#### Release kinetics

*Model- dependent method*. Various kinetic models were used for the evaluation of release patternas mention in below equations [27, 28]:

*First order kinetics*. Explains that the drug release from systems is reliant on concentration, which represents time *vs*. log cumulative percentage drug remaining [27, 28].

$$logQ_{t}=logQ_{0}+k_{1}\frac{t}{2.303}$$ Eq 14

Where Q_t_ is the collective amount of drug release at time t, Q_o_ is the initial concentration of drug, *k*_*1*_ is the First—Order rate constant [29]

*Weibull model*. Weibull model expresses the fraction of drug release (m) in solution at time *t*, by following equation:

$$m=1-exp\left[\frac{-{\left(t-T_{i}\right)}^{b}}{\alpha }\right]$$ Eq 15

or

$$Log\;[-In\;(1–m)]=blog\;(t–T_{i})–log\;\alpha$$

Where, T_*i*_ is the lag time, in various cases zero, α, is the time process and *b* is the shape parameter [30] (b = 1) illustrates the curve as exponential, (b < 1) parabolic with the elevated early slope and after that constant with the exponential, (b > 1) shows S-shaped with increasing curve followed by turning point.

*Hixson–crowell model*. Following equation represents his model:

$$\sqrt[{3}]{Q_{0}}-\sqrt[{3}]{Q_{t}}=k_{HC}t$$ Eq 16

Where Q_0_ = initial concentration of drug, Q_t_ = drug concentration at time *t* and *K*_*HC*_ is the Hixson—Crowell rate constant [28].

*Higuchi model*. Based on diffusion process, the drug release can be described by Higuchi model Equation of Higuchi model is expressed as follows:

Qt=kHt12 Eq 17

Where *k* is the Higuchi release rate constant and *t* is the time in hr [31].

## Results and discussion

The purpose of this study was to prepare fast dispersible tablets of Aceclofenac and to evaluate the effect of different concentrations of avicel PH102 on the compressional behavior of newly developed formulations. The central composite design was used for formulation design. These formulations were arranged and assigned with specific codes for identification purposes and are presented in Table 1.

### Flow properties

Powder blends of all formulations were evaluated for true, bulk and tapped densities and results were found in the range of 1.40–1.47, 0.44–0.52 and 0.51–0.63 g/cm^3^ respectively. Flow properties of all formulations were assessed by using Hausner’s ratio and angle of repose and their respective values were found to be 1.13–1.24, and 33.43–38.31^o^ respectively, indicating better flow properties (Table 2). Formulation blends that failed to meet the acceptable limits of micromeritics characterization and excessive weight than the target formulation were excluded from study (i.e. F11, F12, and F13). Mannitol causes more adhesion problems during compression and this could be overcome by using it in combination with Avicel PH102 which facilitates the compression. After micromeritic evaluation remaining formulations were subjected to compression by direct compression method using manual hydraulic press.

**Table 2:** Micromeritic properties of selected Aceclofenac fast dispersible tablet formulations.

Form Codes	True density ρ_t_ (g/cm^3^)	Bulk density ρ_b_ (g/ cm^3^)	Tapped density ρ_tapp_ (g/ cm^3^)	Hausner's ratio	Angle of repose (θ)
**FA**	1.47	0.46	0.52	1.13	34.53
**FB**	1.40	0.44	0.51	1.17	33.43
**FC**	1.47	0.50	0.60	1.20	38.31
**FD**	1.44	0.49	0.61	1.24	37.09
**FE**	1.46	0.52	0.63	1.23	36.17
**FF**	1.47	0.51	0.59	1.15	34.67

### Disintegration time

All formulations except F13, and F15 showed acceptable disintegration time ranging from 18–45 sec. Formulations F13 and F15 contained 0.5% and 0.1% superdisintegrant respectively, therefore, failed to meet the disintegration time of fast dispersible tablets. Ac-di-sol in the concentration of 1.75% indicated acceptable disintegration time but the formulation F9 having the same amount of superdisintegrant presented disintegration time of 4min which is beyond the requirement of fast dispersible tablets. This difference might be due to the presence of lesser concentration of mannitol (7.63%) which has more water solubility and thus facilitate disintegration process.

On the basis of the weight of tablets closer to target formulation and disintegration time, six formulations were selected for further evaluation. These formulations were assessed by different quality tests and results were found to be inadequate limits. The quality attributes of compressed tablets are mentioned in Table 3.

**Table 3:** Quality attributes of different Aceclofenac fast dispersible tablet formulations at different compressional pressures.

Test parameters	FA	FB	FC	FD	FE	FF
**Weight (mg)**	204.1 ± 1.912	212.1 ± 1.790	207.4 ± 1.955	214.2 ± 2.097	204.3 ± 2.311	212.2 ± 2.529
**Diameter (mm)**	8.468 ± 0.007	8.482 ± 0.010	8.49 ± 0.007	8.47 ± 0.006	8.47 ± 0.021	8.47 ± 0.014
**Thickness (mm)**	2.72 ± 0.055	2.76 ± 0.047	2.72 ± 0.081	2. 77 ± 0.062	2.75 ± 0.087	2.73 ± 0.086
**Hardness (N)**	33.58 ± 8.00	35.40 ± 6.93	34.61 ± 10.46	40.68 ± 9.90	53.42 ± 11.80	43.99 ± 6.25
**Tensile strength (MPa)**	0.932 ± 0.239	0.9628 ± 0.203	0.958 ± 0.311	1.108 ± 0.292	1.468 ± 0.362	1.213 ± 0.207
**Relative density (g/cm**^**3**^**)**	0.93 ± 0.01	0.95 ± 0.01	0.92± 0.02	0.93±0.02	0.92 ± 0.03	0.92 ± 0.02
**Friability (%)**	0.50	0.68	0.30	0.32	0.34	0.29
**Disintegration time (sec)**	18	21	40	36	45	40
**Assay (%)**	99.95 ± 1.34	100.10 ± 0.68	99.63 ± 0.48	100.58 ±1.68	98.63 ± 0.80	99.56 ± 1.23
**Dissolution (%)**	99.42 ± 0.52	100.38 ± 0.71	99.05 ± 0.90	100.35 ± 0.77	99.10 ± 1.04	100.10 ± 1.44

### RSM plot and ANOVA summary

**Fig 1:**
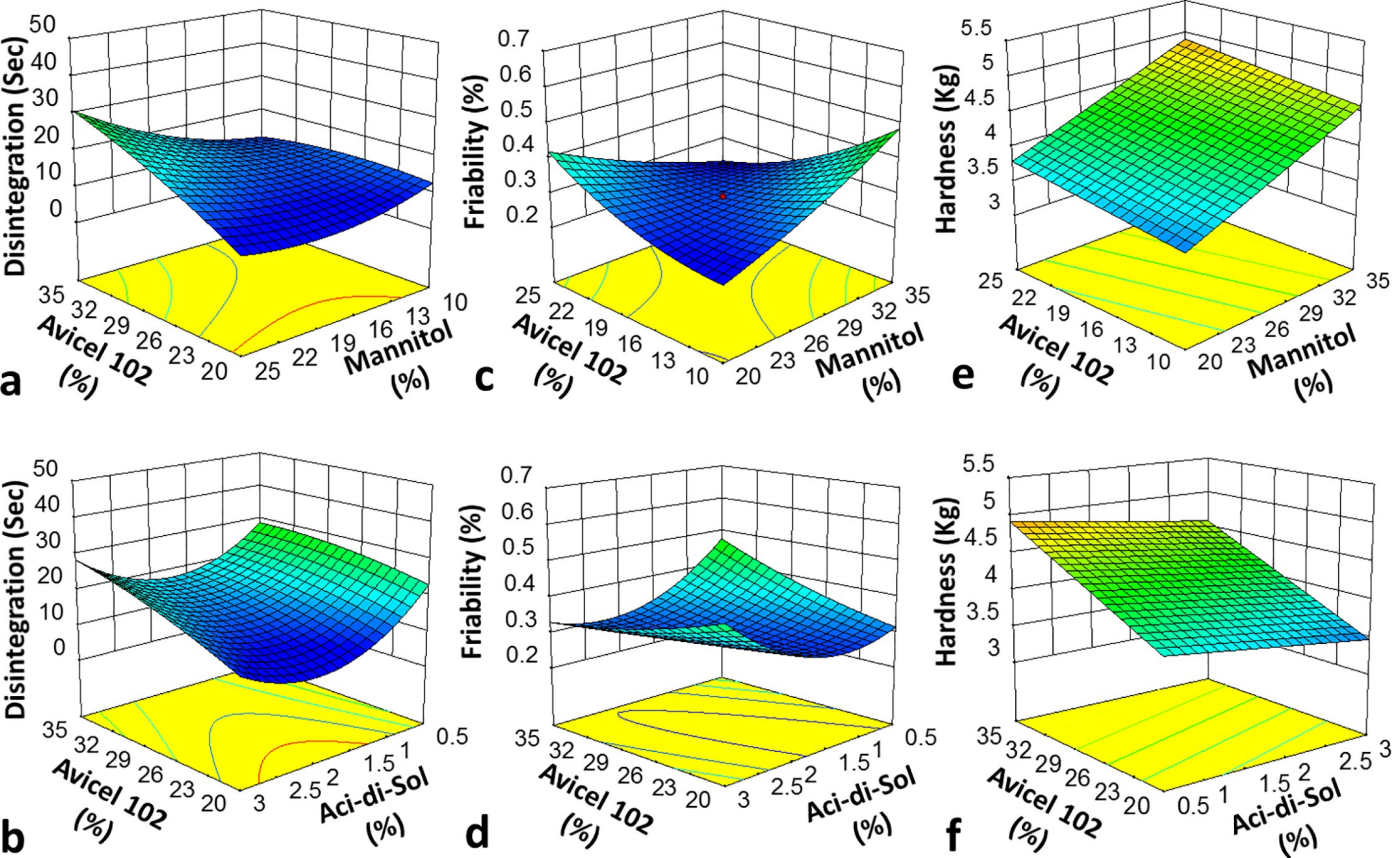
3D Response surface plots of different fast dispersible Aceclofenac tablet formulations presenting effect of independent variables on (a & b) disintegration time, (c & d) Friability and (e & f) Hardness.

It is indicated in the RSM plot that disintegration time was increased with increased concentration of avicel PH102 as shown in Fig 1A and 1B. The ANOVA summary for the first response (disintegration) indicated that the model F value was 4.54 and the Probability value was less than 0.05 indicating that the quadratic model was significant. The “Adeq Precision” was 8.196 which indicated adequate signal-to-noise ratio and the design space could be navigated by the model. RSM plot Fig 1C and 1D indicated that friability was decreased with increased concentration of avicel PH 102 and mannitol. For the second response friability, F value, Probability and Adeq Precision were 10.18, < 0.05 and 11.265 respectively indicating quadratic model was valuable with the satisfactory signal. RSM plot Fig 1E and 1F presented that hardness of fast dispersible aceclofenac tablets was increased with higher concentration of avicel PH102 and mannitol. F value, Probability and Adeq Precision for third response hardness were 10.18, < 0.05 and 11.265 respectively showing model terms and linear model were acceptable with adequate signal. If A = Avicel PH102, B = Mannitol, and C = Ac-di-sol then the final equations in terms of coded factors for disintegration, friability and hardness are given below:

$$Disintegration=+14.01+5.39*A+3.76*B-2.64*C+5.75*AB+2.50*AC-2.50*BC-1.60*A^{2}+3.59*B^{2}+10.81*C^{2}$$

$$Friability=+0.29+0.012*A-0.021*B-4.316E-003*C-0.11*AB-0.069*AC+0.071*BC+0.016*A^{2}+0.039*B^{2}+0.086*C^{2}$$

Hardness=+4.19+0.55*A+0.16*B−0.17*C

### Tensile strength and hardness

Mechanical strength is another important parameter that also represents the inter-particulate bonding. For tablet dosage form it is recommended to determine the mechanical properties of dominant ingredient so as to predict the overall compressional behavior of tablets [32]. Mechanical properties were estimated by measuring the tensile strength which was in the range of 0.932± 0.239–1.468 ± 0.362 MN/m^2^. Tablet hardness of selected formulations was found to be in the range from 33.58 ± 8.00–53.42 ± 11.80N. Results of tensile strength and hardness indicated sufficient mechanical strength of all formulations due to the development of strong inter-particle bonds of powder blends and % friability of the tablets was 0.29–0.68%.

### Compressional behavior analysis

**Fig 2:**
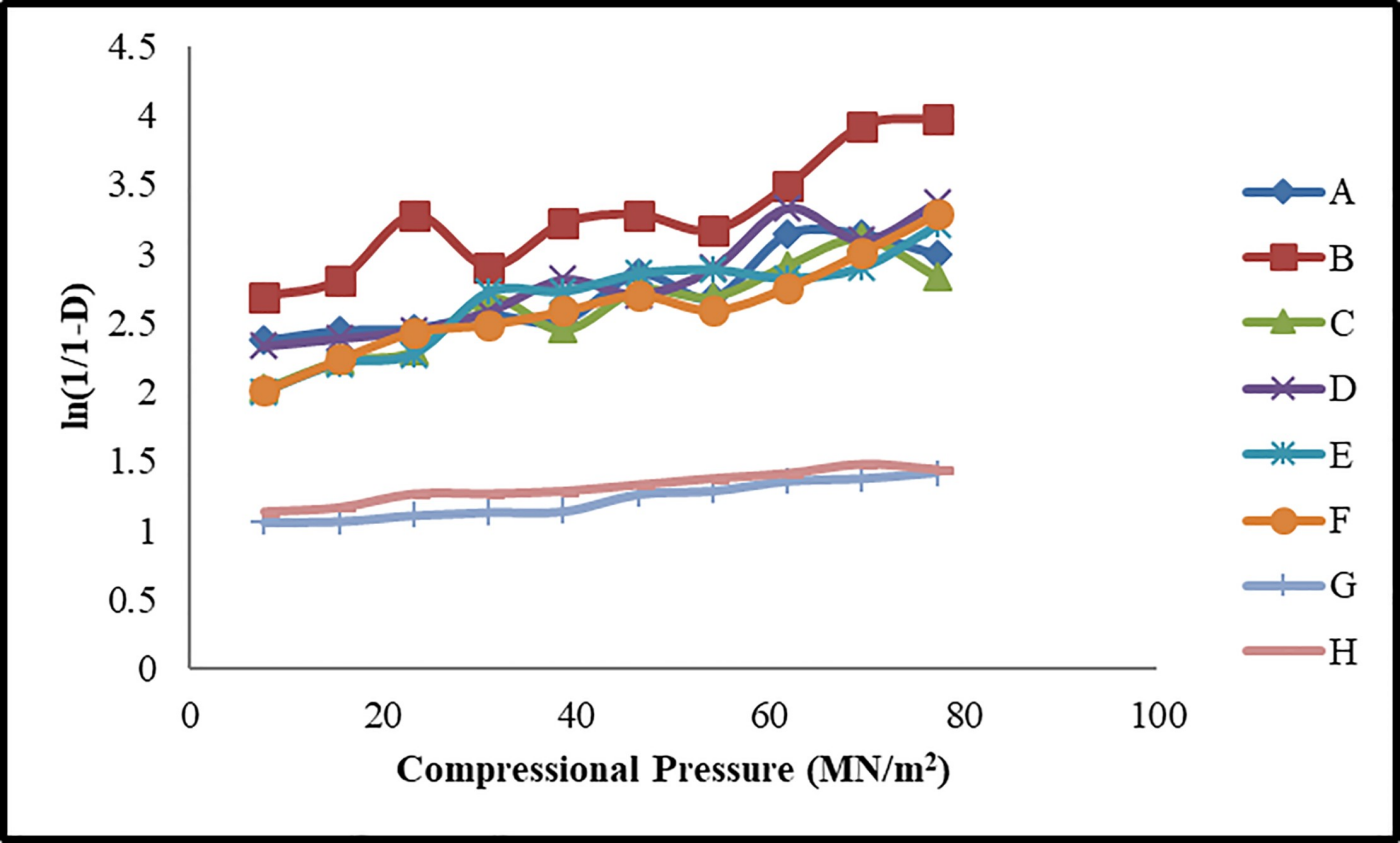
Heckel plots of fast dispersible Aceclofenac tablets.

**Fig 3:**
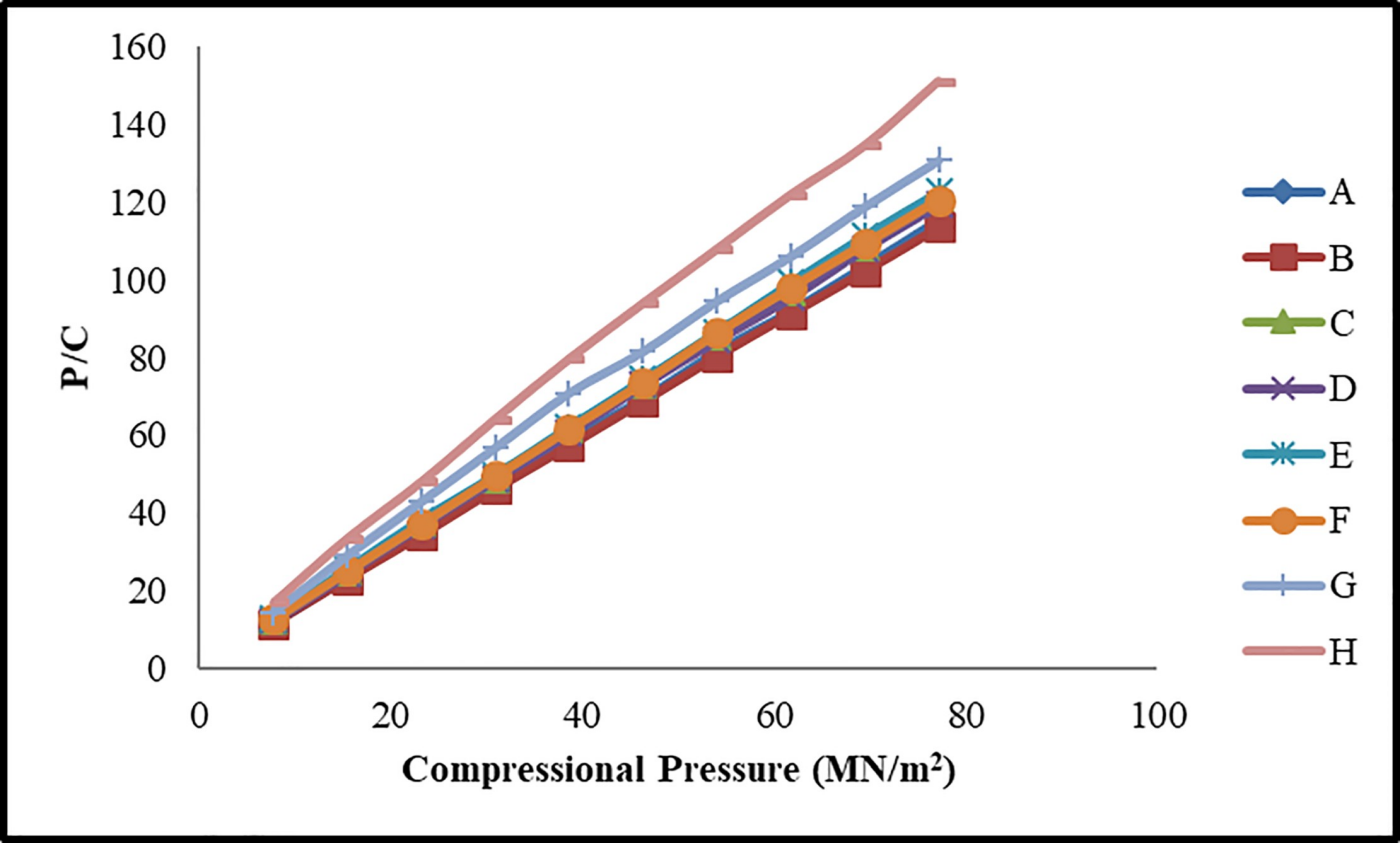
Kawakita plots of fast dispersible Aceclofenac tablets.

For evaluating compressional behavior, ten tablets from each formulation were prepared by applying different compressional pressure 7.72, 23.16, 30.88, 38.0, 46.32, 54.04, 61.76, 69.48 and 77.2 MN/m^2^. Heckel and Kawakita equations were employed for estimating the compressional behavior of fast dispersible Aceclofenac tablets. Previously it was reported that both these compaction equations are suitable for describing the compression process of powder materials based on Avicel PH 102 (*24*). Different parameters of Heckel and Kawakita equations were derived from these plots i.e. D_o,_ D_A,_ D_B,_ P_Y_, Dl and P_K_ and reported in Table 4. Heckel and Kawakita plots are presented in Figs 2 and 3 respectively.

**Table 4:** Compressional parameters obtained from heckel and kawakita equations of formulation blends.

Formulation Codes	Heckel Parameters				Kawakita Parameters	
	D_o_	D_A_	D_B_	P_Y_ (MN/m2)	DI = 1-a	P_k_ = 1/b (MN/m2)
**FA**	0.313	0.892	0.579	86.950	0.325	1.712
**FB**	0.314	0.922	0.608	59.520	0.319	1.040
**FC**	0.343	0.870	0.527	75.750	0.357	4.180
**FD**	0.340	0.883	0.543	64.930	0.349	4.350
**FE**	0.355	0.869	0.514	66.660	0.367	6.560
**FF**	0.339	0.863	0.524	67.560	0.350	7.690

It was reported that D_O_ values for different aceclofenac formulations were increased as the concentration of binder was increased. This phenomenon indicates that at die filling the initial packing of the formulations increases with the higher concentration of binder [32]. In formulations FC, FD, FE and FF, the amount of avicel PH 102 was high (27.5–35%) which resulted in increased hardness, tensile strength and less percentage friability (Table 3). Scientists also found that compaction behavior of powder bed mostly dependent upon the deformation properties of the blends and the applied processing methods [32].

### Heckel analysis

From Heckel plots, it was found that the initial packing of powder blends (D_o_) in FE was found to be high i.e. D_o_ = 0.355 as FE contained increased concentration of avicel PH102 (35%). The D_A_ values at zero and low pressures presented the total degree of packing. Generally formulations with increased concentration of avicel PH102 exhibited the lowest values of D_A_. The D_A_ value which showed the degree of packing at low compressional pressure was found lowest in FE (0.869) and FF (0.863). Values of D_A_ for different formulations observed in the presented sequence: FF<FE<FC<FD<FA<FB.

The D_B_ values presented the densification of powder bed at low pressure which shows the rearrangement of the particles by applying compressional pressure leading to the particle fragmentation. This fragmentation could be plastic or elastic. It was observed that formulations containing low amount of avicel PH102 i.e. FA (0.568) and FB (0.579) showed decreased values of D_B_. The sequence is FE<FF<FC<FD<FA<FB. Amin *et al*., 2012 stated that each powder has its own compressional characteristics. In the form of powder blend these characteristics get significantly changed which affects tablet stability [32]. It was observed that all formulations yielded low values of D_o_ than D_B_ due to particle fragmentation and particle rearrangement in the die at reduced pressure. Generally, increased porosity of powder blend at zero pressure yields low values of D_o._

Another parameter ‘P_Y_’ (mean yield pressure) is the measure of the plasticity of the material. Plastic material is desirable for compression while elastic material creates problems during compression due to elastic recovery. Formulations having greater tendency to deform plastically usually have low values of P_Y_. In this study FB (59.52 MN/m^2^) and FD (64.93 MN/m^2^) showed the lowest ‘P_Y_’ value and the highest plasticity (Table 4). It means that as the amount of avicel PH 102 affects the plasticity of the formulation and facilitates the manufacturing process.

### Kawakita analysis

The Kawakita plot was also constructed to evaluate the compressional behavior of formulations (Fig 2). Kawakita plots presented a linear relationship at different compressional pressures with a correlation coefficient above 99% for formulations FA-FF. From the slope and intercept of Kawakita plots the values of *a* and *ab* were determined respectively. Values of 1-*a* indicated initial relative density (D_I_) of the formulations. By using reciprocal of *b* values, the inverse measurement of plasticity (P_k_) was estimated as given in Table 4. It was observed that initial relative density of formulations (D_I_) was decreased in formulations containing low concentration of avicel PH102. The initial relative density (D_I_) of all formulations was in the range of 0.319–0.367. Results indicated that higher D_I_ values were observed as compared to the corresponding values of D_O_, which has been previously reported by other researches [32]. Furthermore the P_K_ values of these formulations were also higher.

It was observed in the present study that as the concentration of avicel PH102 was increased the P_K_ value was also increased (1.04–7.69 MN/m^2^) which is indicative of elasticity of material. Formulations FE and FF had the highest P_K_ values and FA and FB had the lowest P_K_ values. It was found from Heckel and Kawakita parameters analysis that FE and FF showed the fastest onset of plastic deformation whereas FA and FB showed maximum plastic deformation in combination with aceclofenac and other excipients. However no clear cut variation pattern of Heckel and Kawakita parameters was observed.

### In vitro dissolution

**Fig 4:**
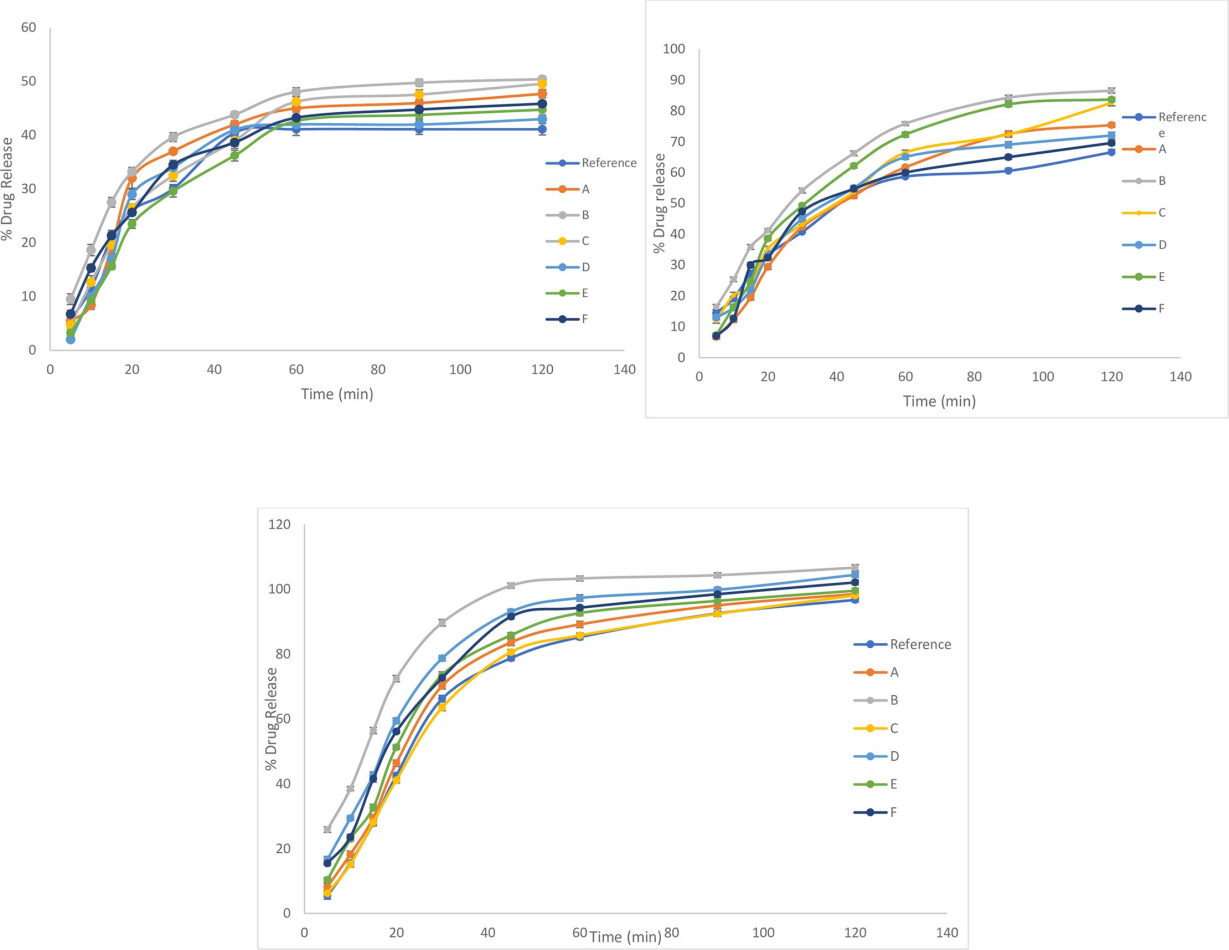
Drug release kinetics of Aceclofenac fast dispersible tablets in 900 ml of (a) pH 1.2 (b) phosphate buffer pH 4.5 (c) phosphate buffer pH 6.8.

In the present study *in vitro* drug release profiles of newly developed and optimized aceclofenac formulations were determined in different dissolution media as shown in Fig 4A–4C. All formulations demonstrated maximum drug release in phosphate buffer pH 6.8. Different kinetic models were used to analyze the release behavior of formulations FA-FF. Results indicated that all formulations followed First-order and Weibull model in different dissolution media with highest *r*^*2*^ values found in phosphate buffer pH 6.8 i.e. 0.946–0.954 and 0.989–0.996 respectively as shown in Table 5.

**Table 5:** Release kinetics of fast dispersible Aceclofenac (100 mg) tablets at different pH.

Formulation Codes	First Order		Higuchi		Hixson-Crowell		Weibull model		
	*r*^2^	K	*r*^2^	K_H_	*r*^2^	K_HC_	*r*^2^	α	Β
		(hr^-1^)		(hr^-1/2^)		(hr^-1/3^)			
0.1N HCI									
**Reference**	0.374	0.007	0.765	4.622	0.645	0.001	0.893	9.695	0.378
**FA**	0.353	0.009	0.768	5.046	0.664	0.001	0.963	8.065	0.367
**FB**	0.408	0.009	0.796	5.107	0.696	0.002	0.939	8.181	0.379
**FC**	0.323	0.009	0.756	5.043	0.653	0.001	0.925	7.764	0.359
**FD**	0.349	0.009	0.760	5.106	0.657	0.001	0.925	7.812	0.364
**FE**	0.324	0.009	0.751	5.031	0.646	0.001	0.919	7.803	0.359
**FF**	0.330	0.009	0.760	5.089	0.659	0.001	0.925	7.745	0.362
**pH 4.5**									
**Reference**	0.681	0.041	0.868	6.480	0.809	0.002	0.950	9.179	0.483
**FA**	0.948	0.021	0.943	8.357	0.944	0.004	0.985	17.192	0.733
**FB**	0.946	0.022	0.945	8.418	0.950	0.004	0.988	17.079	0.736
**FC**	0.950	0.022	0.950	8.391	0.951	0.004	0.991	16.625	0.726
**FD**	0.954	0.022	0.946	8.474	0.950	0.004	0.989	17.511	0.744
**FE**	0.964	0.022	0.953	8.527	0.958	0.005	0.987	20.903	0.785
**FF**	0.950	0.021	0.943	8.359	0.941	0.004	0.982	17.076	0.730
**pH 6.8**									
**Reference**	0.948	0.044	0.781	11.553	0.969	0.012	0.996	41.658	1.267
**FA**	0.946	0.051	0.736	11.438	0.950	0.012	0.991	27.069	1.223
**FB**	0.954	0.052	0.733	11.339	0.951	0.012	0.992	21.981	1.144
**FC**	0.948	0.051	0.713	11.351	0.944	0.012	0.996	29.015	1.248
**FD**	0.952	0.051	0.741	11.398	0.953	0.012	0.993	21.695	1.111
**FE**	0.951	0.050	0.728	11.240	0.948	0.012	0.992	20.114	1.080
**FF**	0.949	0.050	0.735	11.374	0.950	0.012	0.989	24.857	1.165

### Limitation of the study

This study based on the results generated on manual tablet press and not on the large scale rotary compression machine.

## Conclusion

In this study, fast dispersible aceclofenac tablets were prepared and the effect of avicel PH102 was examined on compressional, mechanical and release properties of fast dispersible aceclofenac formulations. Using the Heckel and Kawakita equations, the compressional behavior was observed. The concentration of avicel PH102 exhibited a significant impact on the compressional, mechanical and release properties of the Aceclofenac fast dispersible formulations. Formulation FB having avicel PH102 (20%), mannitol (25%) and ac-di-sol (3%) exhibited excellent compactional strength with rapid disintegration and quick drug release. Hence a suitable selection of excipient with appropriate concentration is important at formulation development stage to ensure stable, elegant, and palatable dosage form for the patient.

## Supporting information

S1 Data (XLSX)

S2 Data (XLSX)
